# 1-(3-Chloro­phen­yl)-2-methyl-4-nitro-1*H*-imidazole-5-carboxamide

**DOI:** 10.1107/S1600536811036609

**Published:** 2011-09-14

**Authors:** Artur Korzański, Paweł Wagner, Maciej Kubicki

**Affiliations:** a Department of Chemistry, Adam Mickiewicz University, Grunwaldzka 6, 60-780 Poznań, Poland; b The ARC Centre of Excellence for Electromaterials Science, Intelligent Polymer Research Institute, University of Wollongong, Innovation Campus, Squires Way, Fairy Meadow, NSW 2519, Australia

## Abstract

In the crystal structure of the title compound, C_11_H_9_ClN_4_O_3_, pairs of N—H⋯N(imidazole) hydrogen bonds connect the mol­ecules into centrosymmetric dimers, which are further connected by N—H⋯O(carbamo­yl) hydrogen bonds into *C*(4) chains along [010]. Inter­play of these two kinds of hydrogen bonds connect the mol­ecules into layers perpendicular to [101]. The imidazole [maximum deviation 0.0069 (9) Å] and phenyl rings are inclined at a dihedral angle of 58.44 (6)°; the nitro group is almost coplanar [dihedral angle 5.8 (2)°] with the imidazole ring while the carbamoyl group is almost perpendicular [70.15 (13)°] to it.

## Related literature

For the synthesis, see: Suwiński *et al.* (1994 ▶). For similar nitro­imidazole derivatives, see: Kubicki (2004*a* ▶,*b* ▶). For a recent experimental charge density study of a nitro­imidazole derivative, see: Paul *et al.* (2011 ▶).
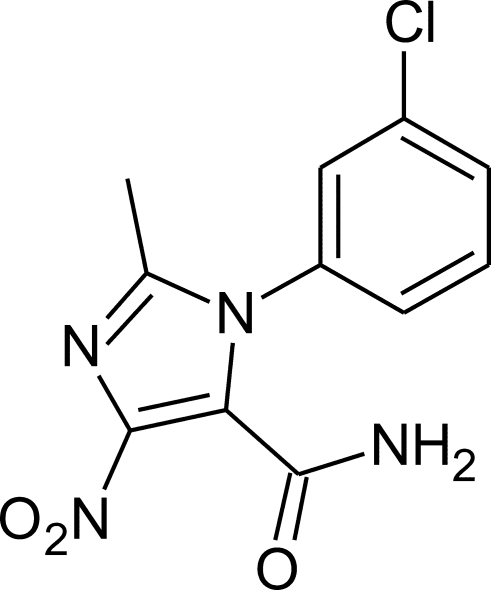


## Experimental

### 

#### Crystal data

C_11_H_9_ClN_4_O_3_
*M* *_r_* = 280.67
Monoclinic, 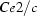

*a* = 21.8417 (14) Å
*b* = 7.3710 (4) Å
*c* = 16.2467 (10) Å
β = 108.680 (7)°
*V* = 2477.9 (3) Å^3^
*Z* = 8
Mo *K*α radiation
μ = 0.32 mm^−1^
*T* = 295 K
0.25 × 0.2 × 0.08 mm

#### Data collection

Agilent Xcalibur Sapphire2 diffractometer
Absorption correction: multi-scan (*CrysAlis PRO*; Agilent, 2010 ▶) *T*_min_ = 0.833, *T*_max_ = 1.000
4943 measured reflections
2702 independent reflections
2185 reflections with *I* > 2σ(*I*)
*R*_int_ = 0.019

#### Refinement

*R*[*F* ^2^ > 2σ(*F* ^2^)] = 0.038
*wR*(*F* ^2^) = 0.104
*S* = 1.04
2702 reflections
197 parameters
H atoms treated by a mixture of independent and constrained refinement
Δρ_max_ = 0.30 e Å^−3^
Δρ_min_ = −0.32 e Å^−3^

### 

Data collection: *CrysAlis PRO* (Agilent, 2010 ▶); cell refinement: *CrysAlis PRO*; data reduction: *CrysAlis PRO*; program(s) used to solve structure: *SIR92* (Altomare *et al.*, 1993 ▶); program(s) used to refine structure: *SHELXL97* (Sheldrick, 2008 ▶); molecular graphics: *SHELXTL* (Sheldrick, 2008 ▶) and *Mercury* (Macrae *et al.*, 2008 ▶); software used to prepare material for publication: *SHELXL97*.

## Supplementary Material

Crystal structure: contains datablock(s) I, global. DOI: 10.1107/S1600536811036609/zk2021sup1.cif

Structure factors: contains datablock(s) I. DOI: 10.1107/S1600536811036609/zk2021Isup2.hkl

Supplementary material file. DOI: 10.1107/S1600536811036609/zk2021Isup3.cml

Additional supplementary materials:  crystallographic information; 3D view; checkCIF report

## Figures and Tables

**Table 1:** Hydrogen-bond geometry (Å, °)

*D*—H⋯*A*	*D*—H	H⋯*A*	*D*⋯*A*	*D*—H⋯*A*
N51—H51*A*⋯N3^i^	0.85 (2)	2.29 (2)	3.130 (2)	169.2 (18)
N51—H51*B*⋯O51^ii^	0.87 (2)	2.03 (2)	2.8938 (19)	171.3 (18)

Symmetry codes: (i) 
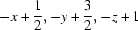
; (ii) 
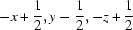
.

**Fig. 1.**
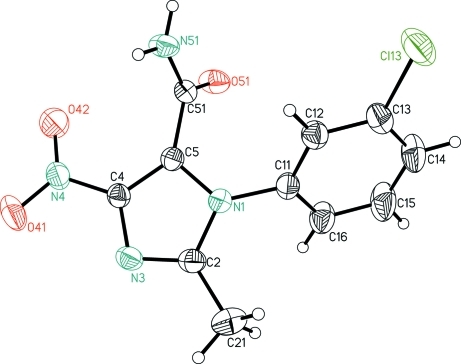
Anisotropic ellipsoid representation of 1 together with atom labelling scheme. The ellipsoids are drawn at 50% probability level, hydrogen atoms are depicted as spheres with arbitrary radii.

**Fig. 2.**
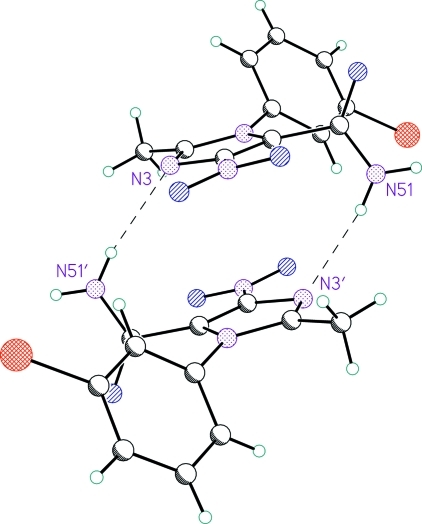
The centrosymmetric dimer formed by N—H···N hydrogen bond; primes denote symmetry code (i) 1/2 - x,3/2 - y,1 - z

**Fig. 3.**
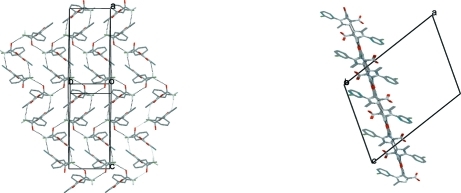
Two mutually perpendicular views of the hydrogen bonded layer of the molecules 1. Neighbouring layers are only loosely bound to one another.

## supplementary crystallographic information

### Comment

In the course of our studies on nitroimidazole derivatives (*e.g.* Kubicki, 2004*a*, 2004*b*; Paul *et al.*, 2011) we have determined the crystal structure of another member of the family of 1-aryl-4-nitro substituted imidazole, 1(3-chlorophenyl)-2-methyl-4-nitro-5-carbamoyl-imidazole (1, Scheme 1).

Fig. 1 shows the perspective view of 1. The two main planar fragments, imidazole (maximum deviation 0.0069 (9) Å) and phenyl rings (0.0125 (13) Å), are inclined by 58.44 (6)°. This value is relatively small: for instance, in three polymorphs of 1-phenyl-2-methyl-4-nitro-5-bromoimidazole (Kubicki, 2004*a*) the twist angle ranges from 86 to 90°, and in 1-(4-chlorophenyl)-2-methyl-4-nitro-1*H*-imidazole-5-carbonitrile (Kubicki, 2004*b*) - 87.5°. The nitro group is nearly coplanar with the imidazole ring (dihedral angle of 5.8 (2)°, while the carbamoyl fragment is, on contrary, almost perpendicular and is inclined by 70.15 (13)° with respect to the imidazole ring plane.

The principal motifs of the crystal sructure are constructed by means of N—H···N and N—H···O hydrogen bonds. N51···N3(1/2 - *x*,3/2 - *y*,1 - *z*) hydrogen bonds connect molecules into centrosymmetric dimers (Fig. 2), and these dimers - the graph set symbol *R*^2^_2_(12) - might be regarded as the building blocks of the structure. The other hydrogen bond, N51···O51(1/2 - *x*,-1/2 + *y*,1/2 - *z*), connect the molecules into C(4) chains along [010] direction. Interplay of these two kinds of hydrogen bonds connect molecules into layers perpendicular to [101], Fig. 3. The neighbouring layers are not connected by any directional intermolecular interactions.

### Experimental

The compound, as an intermediate in purine synthesis, was synthesized by alkaline hydrolysis of 5-cyano derivative in the presence of hydrogen peroxide in good yield (Suwiński *et al.*, 1994).

### Refinement

Hydrogen atoms from methyl group were placed geometrically and refined as riding model with *U*_iso_ set at 1.5 times *U*_eq_ of C21 atom. All other hydrogen atoms were found in the difference Fourier maps and freely refined with isotropic displacement parameters.

### Crystal data

**Table:**

C_11_H_9_ClN_4_O_3_	*F*(000) = 1152
*M**_r_* = 280.67	*D*_x_ = 1.505 Mg m^−^^3^
Monoclinic, *C**e*2/*c*	Mo *K*α radiation, λ = 0.71073 Å
Hall symbol: -C 2yc	Cell parameters from 1970 reflections
*a* = 21.8417 (14) Å	θ = 2.6–27.8°
*b* = 7.3710 (4) Å	µ = 0.32 mm^−^^1^
*c* = 16.2467 (10) Å	*T* = 295 K
β = 108.680 (7)°	Plate, colourless
*V* = 2477.9 (3) Å^3^	0.25 × 0.2 × 0.08 mm
*Z* = 8	

### Data collection

**Table:**

Agilent Xcalibur Sapphire2 diffractometer	2702 independent reflections
Radiation source: Enhance (Mo) X-ray Source	2185 reflections with *I* > 2σ(*I*)
graphite	*R*_int_ = 0.019
Detector resolution: 8.1929 pixels mm^-1^	θ_max_ = 27.9°, θ_min_ = 2.8°
ω–scan	*h* = −28→23
Absorption correction: multi-scan (*CrysAlis PRO*; Agilent, 2010)	*k* = −9→9
*T*_min_ = 0.833, *T*_max_ = 1.000	*l* = −19→20
4943 measured reflections	

### Refinement

**Table:**

Refinement on *F*^2^	Primary atom site location: structure-invariant direct methods
Least-squares matrix: full	Secondary atom site location: difference Fourier map
*R*[*F*^2^ > 2σ(*F*^2^)] = 0.038	Hydrogen site location: inferred from neighbouring sites
*wR*(*F*^2^) = 0.104	H atoms treated by a mixture of independent and constrained refinement
*S* = 1.04	*w* = 1/[σ^2^(*F*_o_^2^) + (0.0514*P*)^2^ + 1.4411*P*] where *P* = (*F*_o_^2^ + 2*F*_c_^2^)/3
2702 reflections	(Δ/σ)_max_ < 0.001
197 parameters	Δρ_max_ = 0.30 e Å^−^^3^
0 restraints	Δρ_min_ = −0.32 e Å^−^^3^

### Special details

**Table:**

Geometry. All e.s.d.'s (except the e.s.d. in the dihedral angle between two l.s. planes) are estimated using the full covariance matrix. The cell e.s.d.'s are taken into account individually in the estimation of e.s.d.'s in distances, angles and torsion angles; correlations between e.s.d.'s in cell parameters are only used when they are defined by crystal symmetry. An approximate (isotropic) treatment of cell e.s.d.'s is used for estimating e.s.d.'s involving l.s. planes.
Refinement. Refinement of *F*^2^ against ALL reflections. The weighted *R*-factor *wR* and goodness of fit *S* are based on *F*^2^, conventional *R*-factors *R* are based on *F*, with *F* set to zero for negative *F*^2^. The threshold expression of *F*^2^ > 2σ(*F*^2^) is used only for calculating *R*-factors(gt) *etc*. and is not relevant to the choice of reflections for refinement. *R*-factors based on *F*^2^ are statistically about twice as large as those based on *F*, and *R*- factors based on ALL data will be even larger.

### Fractional atomic coordinates and isotropic or equivalent isotropic displacement parameters (Å^2^)

**Table:**

	*x*	*y*	*z*	*U*_iso_*/*U*_eq_	
N1	0.17359 (6)	0.85257 (17)	0.33362 (8)	0.0271 (3)	
C11	0.12098 (7)	0.8118 (2)	0.25590 (9)	0.0293 (3)	
C12	0.09423 (8)	0.6397 (2)	0.24501 (11)	0.0332 (4)	
H12	0.1077 (9)	0.552 (3)	0.2894 (12)	0.037 (5)*	
C13	0.04732 (8)	0.6004 (2)	0.16645 (11)	0.0379 (4)	
Cl13	0.01602 (3)	0.38209 (7)	0.14854 (4)	0.05910 (19)	
C14	0.02578 (10)	0.7283 (3)	0.10197 (12)	0.0504 (5)	
H14	−0.0065 (11)	0.699 (3)	0.0509 (15)	0.064 (7)*	
C15	0.05307 (10)	0.8993 (3)	0.11556 (13)	0.0560 (6)	
H15	0.0382 (12)	0.991 (4)	0.0733 (17)	0.072 (7)*	
C16	0.10138 (9)	0.9419 (3)	0.19203 (12)	0.0415 (4)	
H16	0.1217 (10)	1.055 (3)	0.2009 (13)	0.046 (5)*	
C2	0.17800 (8)	0.9965 (2)	0.38931 (10)	0.0305 (3)	
C21	0.12317 (10)	1.1186 (3)	0.38571 (14)	0.0502 (5)	
H21A	0.1342	1.1920	0.4372	0.075*	
H21B	0.0856	1.0473	0.3821	0.075*	
H21C	0.1142	1.1955	0.3355	0.075*	
N3	0.23569 (7)	1.00575 (17)	0.44768 (8)	0.0310 (3)	
C4	0.26864 (7)	0.8643 (2)	0.42808 (9)	0.0266 (3)	
N4	0.33550 (7)	0.83535 (19)	0.47546 (8)	0.0339 (3)	
O41	0.36067 (7)	0.9295 (2)	0.53941 (9)	0.0568 (4)	
O42	0.36375 (6)	0.7157 (2)	0.44992 (9)	0.0543 (4)	
C5	0.23205 (7)	0.76476 (19)	0.35908 (9)	0.0249 (3)	
C51	0.24424 (7)	0.5998 (2)	0.31274 (9)	0.0267 (3)	
O51	0.24669 (7)	0.61445 (15)	0.23864 (7)	0.0387 (3)	
N51	0.24967 (7)	0.44533 (19)	0.35555 (9)	0.0342 (3)	
H51A	0.2520 (9)	0.444 (3)	0.4089 (13)	0.039 (5)*	
H51B	0.2552 (9)	0.346 (3)	0.3297 (12)	0.037 (5)*	

### Atomic displacement parameters (Å^2^)

**Table:**

	*U*^11^	*U*^22^	*U*^33^	*U*^12^	*U*^13^	*U*^23^
N1	0.0274 (6)	0.0263 (6)	0.0261 (6)	0.0018 (5)	0.0067 (5)	−0.0005 (5)
C11	0.0257 (7)	0.0328 (8)	0.0273 (7)	0.0001 (6)	0.0054 (6)	−0.0001 (6)
C12	0.0332 (8)	0.0334 (8)	0.0308 (8)	−0.0010 (7)	0.0071 (7)	0.0005 (7)
C13	0.0336 (9)	0.0394 (9)	0.0379 (9)	−0.0081 (7)	0.0073 (7)	−0.0065 (7)
Cl13	0.0610 (3)	0.0487 (3)	0.0575 (3)	−0.0197 (2)	0.0048 (3)	−0.0114 (2)
C14	0.0399 (10)	0.0630 (13)	0.0362 (10)	−0.0071 (9)	−0.0046 (8)	0.0027 (9)
C15	0.0496 (12)	0.0587 (13)	0.0426 (10)	−0.0043 (10)	−0.0093 (9)	0.0181 (10)
C16	0.0367 (9)	0.0388 (10)	0.0409 (9)	−0.0040 (8)	0.0012 (7)	0.0092 (8)
C2	0.0370 (8)	0.0255 (7)	0.0299 (7)	0.0026 (6)	0.0118 (6)	−0.0003 (6)
C21	0.0482 (11)	0.0447 (11)	0.0579 (12)	0.0155 (9)	0.0174 (9)	−0.0052 (9)
N3	0.0387 (7)	0.0269 (7)	0.0272 (6)	0.0012 (6)	0.0103 (6)	−0.0035 (5)
C4	0.0303 (8)	0.0266 (7)	0.0215 (6)	0.0000 (6)	0.0066 (6)	0.0000 (6)
N4	0.0332 (7)	0.0368 (7)	0.0279 (6)	−0.0020 (6)	0.0045 (6)	−0.0023 (6)
O41	0.0461 (8)	0.0674 (9)	0.0430 (7)	−0.0046 (7)	−0.0050 (6)	−0.0219 (7)
O42	0.0351 (7)	0.0607 (9)	0.0597 (8)	0.0109 (6)	0.0049 (6)	−0.0182 (7)
C5	0.0278 (7)	0.0243 (7)	0.0219 (6)	0.0008 (6)	0.0071 (6)	0.0016 (5)
C51	0.0283 (7)	0.0257 (7)	0.0245 (7)	0.0003 (6)	0.0062 (6)	−0.0027 (6)
O51	0.0627 (8)	0.0305 (6)	0.0268 (6)	0.0056 (6)	0.0196 (5)	0.0008 (5)
N51	0.0530 (9)	0.0239 (7)	0.0266 (7)	0.0029 (6)	0.0142 (6)	−0.0014 (6)

### Geometric parameters (Å, °)

**Table:**

N1—C5	1.3719 (19)	C2—C21	1.484 (2)
N1—C2	1.3774 (19)	C21—H21A	0.9600
N1—C11	1.4408 (19)	C21—H21B	0.9600
C11—C16	1.377 (2)	C21—H21C	0.9600
C11—C12	1.384 (2)	N3—C4	1.361 (2)
C12—C13	1.388 (2)	C4—C5	1.364 (2)
C12—H12	0.94 (2)	C4—N4	1.432 (2)
C13—C14	1.376 (3)	N4—O42	1.2228 (19)
C13—Cl13	1.7360 (18)	N4—O41	1.2236 (18)
C14—C15	1.381 (3)	C5—C51	1.498 (2)
C14—H14	0.93 (2)	C51—O51	1.2270 (18)
C15—C16	1.384 (3)	C51—N51	1.319 (2)
C15—H15	0.94 (3)	N51—H51A	0.85 (2)
C16—H16	0.93 (2)	N51—H51B	0.87 (2)
C2—N3	1.314 (2)		
			
C5—N1—C2	107.62 (12)	N1—C2—C21	123.73 (15)
C5—N1—C11	124.74 (12)	C2—C21—H21A	109.5
C2—N1—C11	127.22 (13)	C2—C21—H21B	109.5
C16—C11—C12	121.72 (15)	H21A—C21—H21B	109.5
C16—C11—N1	118.89 (15)	C2—C21—H21C	109.5
C12—C11—N1	119.27 (14)	H21A—C21—H21C	109.5
C11—C12—C13	117.78 (15)	H21B—C21—H21C	109.5
C11—C12—H12	121.0 (11)	C2—N3—C4	104.35 (13)
C13—C12—H12	121.2 (11)	N3—C4—C5	112.95 (14)
C14—C13—C12	121.94 (17)	N3—C4—N4	120.85 (13)
C14—C13—Cl13	119.18 (14)	C5—C4—N4	126.15 (14)
C12—C13—Cl13	118.87 (14)	O42—N4—O41	123.99 (15)
C13—C14—C15	118.62 (17)	O42—N4—C4	117.62 (13)
C13—C14—H14	119.8 (15)	O41—N4—C4	118.38 (14)
C15—C14—H14	121.6 (15)	C4—C5—N1	103.76 (13)
C14—C15—C16	121.11 (18)	C4—C5—C51	134.27 (14)
C14—C15—H15	120.4 (15)	N1—C5—C51	121.97 (13)
C16—C15—H15	118.5 (15)	O51—C51—N51	124.64 (14)
C11—C16—C15	118.79 (18)	O51—C51—C5	119.48 (13)
C11—C16—H16	119.2 (13)	N51—C51—C5	115.84 (13)
C15—C16—H16	122.0 (12)	C51—N51—H51A	121.0 (14)
N3—C2—N1	111.31 (14)	C51—N51—H51B	118.4 (12)
N3—C2—C21	124.90 (15)	H51A—N51—H51B	120.2 (19)
			
C5—N1—C11—C16	−115.99 (18)	C21—C2—N3—C4	177.36 (16)
C2—N1—C11—C16	55.6 (2)	C2—N3—C4—C5	−0.99 (17)
C5—N1—C11—C12	60.0 (2)	C2—N3—C4—N4	176.64 (14)
C2—N1—C11—C12	−128.44 (17)	N3—C4—N4—O42	−173.81 (15)
C16—C11—C12—C13	1.1 (3)	C5—C4—N4—O42	3.5 (2)
N1—C11—C12—C13	−174.80 (15)	N3—C4—N4—O41	7.2 (2)
C11—C12—C13—C14	−2.3 (3)	C5—C4—N4—O41	−175.54 (15)
C11—C12—C13—Cl13	176.52 (12)	N3—C4—C5—N1	1.33 (17)
C12—C13—C14—C15	1.6 (3)	N4—C4—C5—N1	−176.15 (14)
Cl13—C13—C14—C15	−177.23 (17)	N3—C4—C5—C51	−179.51 (15)
C13—C14—C15—C16	0.4 (3)	N4—C4—C5—C51	3.0 (3)
C12—C11—C16—C15	0.8 (3)	C2—N1—C5—C4	−1.11 (15)
N1—C11—C16—C15	176.73 (17)	C11—N1—C5—C4	171.84 (13)
C14—C15—C16—C11	−1.6 (3)	C2—N1—C5—C51	179.59 (13)
C5—N1—C2—N3	0.58 (17)	C11—N1—C5—C51	−7.5 (2)
C11—N1—C2—N3	−172.15 (14)	C4—C5—C51—O51	−110.4 (2)
C5—N1—C2—C21	−176.59 (16)	N1—C5—C51—O51	68.6 (2)
C11—N1—C2—C21	10.7 (2)	C4—C5—C51—N51	71.6 (2)
N1—C2—N3—C4	0.23 (17)	N1—C5—C51—N51	−109.36 (17)

### Hydrogen-bond geometry (Å, °)

**Table:**

*D*—H···*A*	*D*—H	H···*A*	*D*···*A*	*D*—H···*A*
N51—H51A···N3^i^	0.85 (2)	2.29 (2)	3.130 (2)	169.2 (18)
N51—H51B···O51^ii^	0.87 (2)	2.03 (2)	2.8938 (19)	171.3 (18)

Symmetry codes: (i) −*x*+1/2, −*y*+3/2, −*z*+1; (ii) −*x*+1/2, *y*−1/2, −*z*+1/2.
